# IFN-γ and TNF-α Synergize to Inhibit CTGF Expression in Human Lung Endothelial Cells

**DOI:** 10.1371/journal.pone.0045430

**Published:** 2012-09-20

**Authors:** Roderich Laug, Markus Fehrholz, Norbert Schütze, Boris W. Kramer, Vera Krump-Konvalinkova, Christian P. Speer, Steffen Kunzmann

**Affiliations:** 1 Orthopedic Center for Musculoskeletal Research, Molecular Orthopedics, University of Würzburg, Würzburg, Germany; 2 Children`s Hospital, University of Würzburg, Würzburg, Germany; 3 Department of Pediatrics, University Hospital Maastricht, Maastricht, The Netherlands; 4 Institute for Prevention of Cardiovascular Diseases, University of Munich, Munich, Germany; National Cancer Institute, United States of America

## Abstract

Connective tissue growth factor (CTGF/CCN2) is an angiogenetic and profibrotic factor, acting downstream of TGF-β, involved in both airway- and vascular remodeling. While the T-helper 1 (Th1) cytokine interferon-gamma (IFN-γ) is well characterized as immune-modulatory and anti-fibrotic cytokine, the role of IFN-γ in lung endothelial cells (LEC) is less defined. Tumour necrosis factor alpha (TNF-α) is another mediator that drives vascular remodeling in inflammation by influencing CTGF expression. In the present study we investigated the influence of IFN-γ and TNF-α on CTGF expression in human LEC (HPMEC-ST1.6R) and the effect of CTGF knock down on human LEC. IFN-γ and TNF-α down-regulated CTGF in human LEC at the promoter-, transcriptional- and translational-level in a dose- and time-dependent manner. The inhibitory effect of IFN-γ on CTGF-expression could be almost completely compensated by the Jak inhibitor AG-490, showing the involvement of the Jak-Stat signaling pathway. Besides the inhibitory effect of IFN-γ and TNF-α alone on CTGF expression and LEC proliferation, these cytokines had an additive inhibitory effect on proliferation as well as on CTGF expression when administered together. To study the functional role of CTGF in LEC, endogenous CTGF expression was down-regulated by a lentiviral system. CTGF silencing in LEC by transduction of CTGF shRNA reduced cell proliferation, but did not influence the anti-proliferative effect of IFN-γ and TNF-α. In conclusion, our data demonstrated that CTGF was negatively regulated by IFN-γ in LEC in a Jak/Stat signaling pathway-dependent manner. In addition, an additive effect of IFN-γ and TNF-α on inhibition of CTGF expression and cell proliferation could be found. The inverse correlation between IFN-γ and CTGF expression in LEC could mean that screwing the Th2 response to a Th1 response with an additional IFN-γ production might be beneficial to avoid airway remodeling in asthma.

## Introduction

Although asthma is considered to be an entirely reversible disorder, patients with asthma experience an accelerated rate of deterioration of the respiratory function [1]. This deterioration is clinically characterised by irreversible or only partially reversible airway obstruction and persistent airway hyperresponsiveness. Recently, attention has been focused on the structural changes in the airways, collectively referred to as airway remodeling. These changes include thickening of the basement membrane, airway smooth muscle (ASM) hypertrophy and/or hyperplasia, alterations in the composition of the extracellular matrix (ECM), lung fibroblast proliferation and vascular remodeling [2]. The last one, including angiogenesis, vasodilatation, and microvascular leakage, is one of the most prominent and uniform findings in asthma [2]. Compared with control subjects, the total number of vessels and vascular areas in the airways are increased in asthmatics [3]. This increased number and size of vessels and the vascular leakage contribute to the thickening of the airway wall, resulting in a narrowing of the lumen. In addition, individuals with stable asthma have shown increased levels of circulating bone marrow-derived endothelial progenitor cells (EPC) in their blood [4].

TGF-β is one of the critical mediators in the pathogenesis of asthma, involved in the regulation of both airway inflammation and airway/vascular remodeling [5]. Increased TGF-β levels were detected in asthmatic bronchoalveolar lavage fluids [6]. TGF-β acts as suppressive cytokine that prevents lymphocyte activation and mediates peripheral tolerance toward allergens [5]. In addition, TGF-β assists during wound repair inducing ECM deposition, and thus is critically contributing to lung fibrosis [5]. Furthermore, dysregulation of TGF-β is associated with an increasing number of vascular pathologies [7]. Downstream of TGF-β, connective tissue growth factor (CTGF) modulates the cellular response to TGF-β and contributes to wound healing and fibrotic response [8]. Furthermore, CTGF regulates angiogenesis and endothelial cell function, especially by facilitating the angiogenetic response that supports tissue repair [9]. The role of CTGF in airway remodeling in asthma has not been elucidated in detail, but CTGF has the potential to be a key factor in the development and maintenance of the structural changes associated with severe persistent asthma due to its simultaneous angiogenic and fibrogenic properties [10]–[12].

The role of the Th1 cytokine IFN-γ in asthma is controversial. On the one hand, the level of IFN-γ was decreased in asthma [13], [14] and the potential use of IFN-γ in allergic diseases has been suggested in a number of human and animal studies [15]–[18]. Loss of IFN-γ action, therefore, was suggested to contribute to the altered immune state characterising asthma. On the other hand, elevated IFN-γ has been also identified in asthmatic patients, suggesting that Th1-like cells may also contribute to, rather than inhibit, the asthma physiopathology [19], [20]. Furthermore, in an allergen-challenge asthma model, bone marrow-derived endothelial progenitor cells (EPC) recruitment was Th1 and Th2 dependent and associated with increased microvessel density [4]. In addition, both Th1 and Th2 cytokines can modulate neo-vascularization by controlling EC function, including migration, invasion, proliferation, and apoptosis, all of which are crucial for the formation of new blood vessels [21], [22]. This observation is in agreement with the hypothesis that the Th1/Th2 paradigm could be much more complex than what was initially appreciated [23]. The Th1/Th2 paradigm suggested that Th1 and Th2 cells counterbalance each other and that Th1 cells protect or prevent Th2-mediated allergic disease and asthma [24]. However, antigen specific Th1 cells were ineffective in reducing airway hyperreactivity induced by Th2 cells and caused serious airway inflammation [23]. The possible role and mechanisms of IFN-γ-induced vascular remodelling in lung endothelial cells (LEC) have not been adequately defined yet.

The IFN-γ-induced Stat-signalling pathway is well characterised [25]: IFN-γ binds to its receptor (IFN-γR) and leads to the aggregation of α- and β-chains, constitutively associated with Janus Kinases (JAKs). Once activated, JAKs phosphorylate the IFN-γRα chain creating a docking site for Stat1, which is then phosphorylated and associated in homodimers. The p-Stat1 homodimer translocates to the nucleus where it is able to bind to specific DNA sequences. Stat1 phosphorylation is necessary for Stat1 dimerization, nuclear translocation, and DNA binding [25].

TNF-α is another cytokine involved in inflammation, triggering vascular remodeling processes [26]. Increased expression of TNF-α was reported in severe and refractory asthma [27], [28] and TNF-α can inhibit CTGF expression [29]–[31].

The current study was designed to address the question of whether the anti-fibrotic and angiostatic cytokine IFN-γ was capable to modulate CTGF production in airway endothelial cells and how this potential regulation is influenced by TNF-α. In addition, the signaling mechanisms by which IFN-γ modulates CTGF production and the effects of CTGF gene silencing on LEC were explored.

## Methods

### Reagents

Recombinant TGF-β1, TNF-α, and IFN-γ were obtained from R&D Systems (Abingdon, UK). The Jak-Inhibitor (AG-490) was obtained from Calbiochem (658411; La Jolla, CA, USA), rCTGF from Biovendor (Heidelberg, Germany). The Cell Proliferation ELISA (BrdU, chemiluminescent) and protease inhibitor cocktail tablets were provided by Roche (Mannheim, Germany).

### Cell Culture

HPMEC-ST1.6R, a human LEC line, has already been described and exhibited a broad spectrum of primary human microvascular endothelial cell characteristics *in vitro* [32], [33]. Cells were grown on plastic tissue culture dishes in RPMI medium containing 10% fetal bovine serum (experiments with TGF-β1 were done in serum-free medium), penicillin (100 U/ml), streptomycin (100 µg/ml), and amphotericin B (0.25 µg/ml). On 6-cm dishes, cells were allowed to grow until 80% confluence before adding the investigated agents. Incubation was carried out at 37°C in a humidified atmosphere of 95% room air and 5% CO_2_. HPMEC-ST1.6R cells were used in experiments between passage number 3 and 34. HEK 293 T cells, human embryonic kidney cells, were obtained from ATCC (Manassas, USA). Cells were cultured in DMEM Ham`s F12 medium with L-Glutamin, containing 10% fetal bovine serum, 1% penicillin (100 U/ml) and streptomycin (100 µg/ml).

### Viability Detection

HPMEC-ST1.6R cell viability after exposure to TGF-β1 (10 ng/ml), TNF-α (20 ng/ml), IFN-γ (500 U/ml), rCTGF (200 ng/ml), or the Jak-inhibitor AG-490 (30 µM) was evaluated after 1–3 days using the flow cytometry–based quantification of ethidium bromide (1 mmol/L; Sigma Aldrich) uptake.

### Proliferation Assay

To monitor DNA synthesis, HPMEC-ST1.6R cells were seeded in 24-well plates (Costar, Corning, NY) and allowed to attach for 24 h, followed by serum starvation for 24 h (experiments with TGF-β1). Cells (triplicates) were then incubated with rCTGF, IFN-γ, TNF-α, TGF-β1 alone or in combination at different time points with the simultaneous addition of BrdU. For the analysis of cell proliferation the chemiluminescent BrdU ELISA kit from Roche was used according to the manufacturer’s protocol. The signals were visualised and evaluated on a LumiImager work station using image analysis software (Boehringer, Mannheim, Germany).

### Transfections and Reporter Gene Assays

Cloning of the CTGF promoter (pCT-sb)-Luciferase plasmid has already been described elsewhere [34]. pCT-sb (2 µg) and *Renilla* luciferase control reporter vector (phRL-TK; 5 ng) were transfected with LipofectaminPlus (Invitrogen) into HPMEC-ST1.6R and seeded into 6-well plates. Transfection medium (Optimem-medium, Invitrogen) was changed to 0.2% fetal bovine serum after 2 hours. Twenty-four hours after transfection, cells were treated with indicated molecules. After 16 hours, luciferase activity was measured by the dual luciferase assay system (Promega Biotech Inc., Madison, Wis) according to the manufacturer’s instruction using a Berthold MiniLumat LB 9506 luminometer (Bad Wildbach, Germany). Firefly luciferase activity was normalised by the activity of *Renilla* luciferase under the control of thymidine kinase promoter of phRL-TK. Results are given as relative light units. All values were obtained from experiments carried out in triplicates and repeated at least 3 times.

### RNA Extraction and Reverse Transcription

Total RNA was isolated from HPMEC-ST1.6R cells using the RNeasy mini kit (Qiagen, Hilden, Germany) according to the manufacturer’s protocol. The RNA was eluted in 40 µL water and subjected to reverse transcription. Purity and yield of the RNA were photometrically determined. Overall, 5 µg total RNA was reverse transcribed by addition of 500 µg/mL oligo (dT)12 primers (Roche, Basel, Switzerland), RNAse Inhibitor (10 U/µL, Roche), dNTP (5 mmol/L each dNTP; Qiagen), and Omniscript transcriptase (Qiagen) (0.2 U/µL) for 1 hour at 37°C. The cDNA was denatured at 90°C for 5 minutes and used for PCR amplification.

### Real-time PCR Analysis

The CTGF or β-actin quantitative real-time PCR (TaqMan™) primers and probes were obtained from Applied Biosystems (Weiterstadt, Germany). All PCRs were performed utilizing 1 µg/µl cDNA per reaction in dublicates of 30 µl volume on an ABI Prism 7300 Sequence Detection System (TaqMan), using a 2-step PCR protocol after initial denaturing of the DNA (10 min at 95°C) with 40 cycles of 95°C for 15 s and 60°C for 1 min. A universal master mix, obtained from Applied Biosystems, included all reagents as Taq-polymerase and buffer apart from specific primers and probes. All amplification batches included no template controls. Neither negative controls nor mRNA probes led to elevated fluorescence signals after PCR. Dilution experiments were performed to ensure similar efficiency of the PCRs, and standard curves were calculated referring the threshold cycle to the log of each cDNA dilution step. Results of CTGF or transgelin were normalized to β-actin and mean fold changes in mRNA expression were calculated by the ΔΔCt-method [35].

### Western Blot Analysis

HPMEC-ST1.6R cells were rinsed with ice-cold tris-buffered saline (TBS) and incubated in 100 µl lysis buffer (Cell lysis buffer, Cell Signaling Technologies, MA, USA, Complete Mini protease inhibitor cocktail tablets and PhosStop phosphatase inhibitor cocktail tablets (Roche, Germany; 0.1 mM PMSF, Merck KGaA, Germany)) for 10 minutes on ice. The lysate was cleared by centrifugation with 30,000 g for 10 min, and the supernatant was used for Western immunoblotting analysis. Protein concentrations were determined for each sample using the Bradford assay (Bio-Rad, Richmond, CA, USA) and equal amounts of cellular protein were loaded and separated by SDS-PAGE on 10% to 12% Bis-Tris gels and electrophoretically transferred to polyvinylidene difluoride or nitrocellulose blotting membranes (Amersham Pharmacia Biotech, Piscataway, NJ, USA). Membranes were blocked in 5% BSA for 1 hour at room temperature and successively incubated with primary antibodies overnight at 4°C. Western blots were probed with primary antibodies to β-actin, CTGF (Santa Cruz Biotechnology, CA, USA), Stat-1, phospho-Stat1 (Tyr705, Cell Signaling Technologies, MA, USA), and TGF-ß1 (R&D Systems GmbH, Wiesbaden-Nordenstadt, Germany and Sigma-Aldrich Chemie GmbH, Steinheim, Germany) followed by the corresponding horseradish peroxidase-conjugated secondary antibody (Pierce, Bonn, Germany) for 1 h at room temperature. Specific protein bands were visualised using enhanced chemiluminescence (SuperSignal West Dura, Pierce Inc., Rockford, USA; GE Healthcare Europe GmbH, München, Germany) and detected using the LAS 3000 computer-based luminescent image analyzer (FujiFilm, Tokyo, Japan). Accumulated signals were analyzed using AIDA software (Raytest, Germany).

### CTGF Gene Silencing by Lentiviral Transduction

For the downregulation of endogenous CTGF expression in HPMEC-ST1.6R, a lentiviral system (psPAX2, pMD2G, Addgene Inc., Cambridge, USA) with a set of different shRNAs (Open Biosystems, Inc., Fisher Scientific GmbH, Schwerte, Germany) was used. Scrambled shRNA, vector only (Addgene Inc., Cambridge, USA), and GFP (Sigma-Aldrich Chemie GmbH, Steinheim, Germany) were used for controls. The first day 6×10^5^ HEK 293T cells (ATCC, Manassas, USA) were seeded per 6-well in DMEM/Hams F12 cell culture medium (PAA Laboratories, Pasching, Austria) containing 10% FCS (PAA) and 0,1% Penicillin/Streptomycin (PAA). After 24 h of incubation the cells reached about 80% confluency and were transfected. Lipofectamin 2000™ transfection reagent (Invitrogen GmbH, Darmstadt, Germany) and DMEM/Hams F12 medium, without additives, where then mixed and incubated for 5 minutes at room temperature. In the next step, psPAX2, pMD2.G and shRNA respectively scrambled, vector only and GFP plasmid were mixed and given to the Lipofectamin 2000™/DMEM Hams F12 mixture. After 20 minutes of incubation at room temperature, HEK 293 T cells were transfected by this mixture and incubated for 18 h at 37°C. On day three, medium was changed with DMEM Hams F12 containing 30% FCS and 1% Penicillin/Streptomycin, incubated for further 24 h at 37°C, and then replaced with normal growth medium. 1×10^5^ HPMEC-ST1.6R cells were seeded per 6-well for later transduction. The next day, the virus supernatant of the HEK 293T cells was centrifuged and given, together with Polybrene (Sigma-Aldrich Chemie GmbH, Steinheim, Germany), to the target cells. The transduced target cells were incubated for further 24 h at 37°C and then medium was replaced with normal growth medium. Two to three days after lentiviral transduction the effect could be observed under UV light by green fluorescing GFP control.

### Statistical Analysis

All results shown are representative of three separate experiments. Results are given as means ± SEM. Data were analyzed by the Mann-Whitney-Wilcoxon test. A p-value <0.05 was considered significant. All statistical analyses were performed using the statistical software GraphPad Prism 5.0.

## Results

### Effect of CTGF on LEC Proliferation

**Figure 1:**
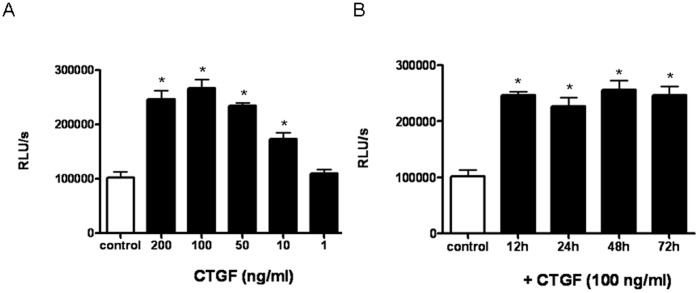
Effect of rCTGF on proliferation of LEC. **A.** Proliferation in LEC was assessed 3 days after the addition of rCTGF at the indicated concentration. **B.** Proliferation in LEC was assessed after the addition of 100 ng/ml CTGF at the indicated time points. Values are means ± SEMs for 3 replicate experiments. Significant differences (p<0.05) compared with untreated cells are marked by *. RLU/s (relative light unit/second).

To investigate the influence of CTGF to the proliferative behaviour of LEC, the effect of rCTGF on the *in vitro* proliferation of human LEC was analysed. CTGF induced LEC proliferation in a dose-dependent manner (Figure 1A). The highest increase in proliferation (2.6-fold) was measured with a concentration of 100 ng/ml of rCTGF. There was no difference of cell proliferation in LEC treated with 100 ng/ml rCTGF between different time points (12 h–72 h; Figure 1B).

### Regulation of CTGF Expression by IFN-γ in LEC

**Figure 2:**
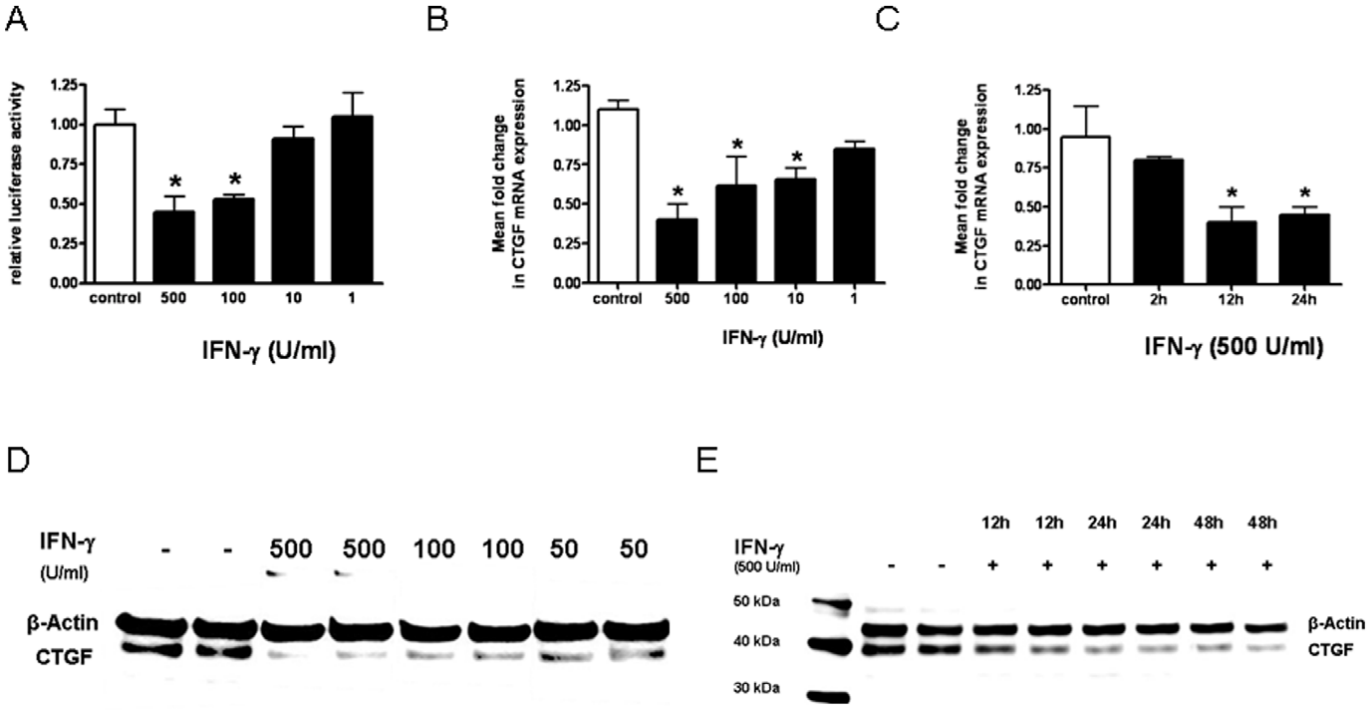
Effect of IFN-γ on CTGF expression in LEC. **A.** Luminometric analysis of CTGF-Luc reporter transfected LEC: LEC were incubated with medium alone (control) or with decreasing concentrations of IFN-γ. * p<0.05 vs medium alone. **B+C.** CTGF mRNA expression: LEC were incubated with different concentrations of IFN-γ for 12 h (B) or with IFN-γ (500 U/ml) for various duration (C). mRNA expression of CTGF normalized for β-actin were measured by real-time PCR. Significant differences (p<0.05) compared with untreated cells are marked by *. **D+E.** CTGF protein expression: LEC were incubated with different concentrations of IFN-γ (D) and for different time points (E). CTGF and β-actin expression was detected by immunoblotting. A representative of 3 independent experiments is shown.

We examined the effect of IFN-γ on CTGF promoter activity in LEC. IFN-γ significantly reduced CTGF promoter activity in a dose dependent manner (Figure 2A). The maximum inhibition of luciferase activity was a 2.2-fold reduction with 500 U/ml IFN-γ. IFN-γ decreased CTGF mRNA expression in a dose-dependent (Figure 2B) and time-dependent (Figure 2C) manner at the transcriptional-level. A maximum inhibition of CTGF mRNA by IFN-γ was observed after 12 h (2.5-fold inhibition). At the translation-level, IFN-γ decreased CTGF protein expression in LEC in a dose- (Figure 2D) and time-dependent manner (Figure 2E) with a maximal inhibition after 48 h with an IFN-γ concentration of 500 U/ml. These results indicated that IFN-γ down-regulated CTGF at the promoter-, transcriptional- and translation-level in LEC.

### Down-regulation of CTGF by IFN-γ is Mediated by Jak/Stat Signaling Pathway

**Figure 3:**
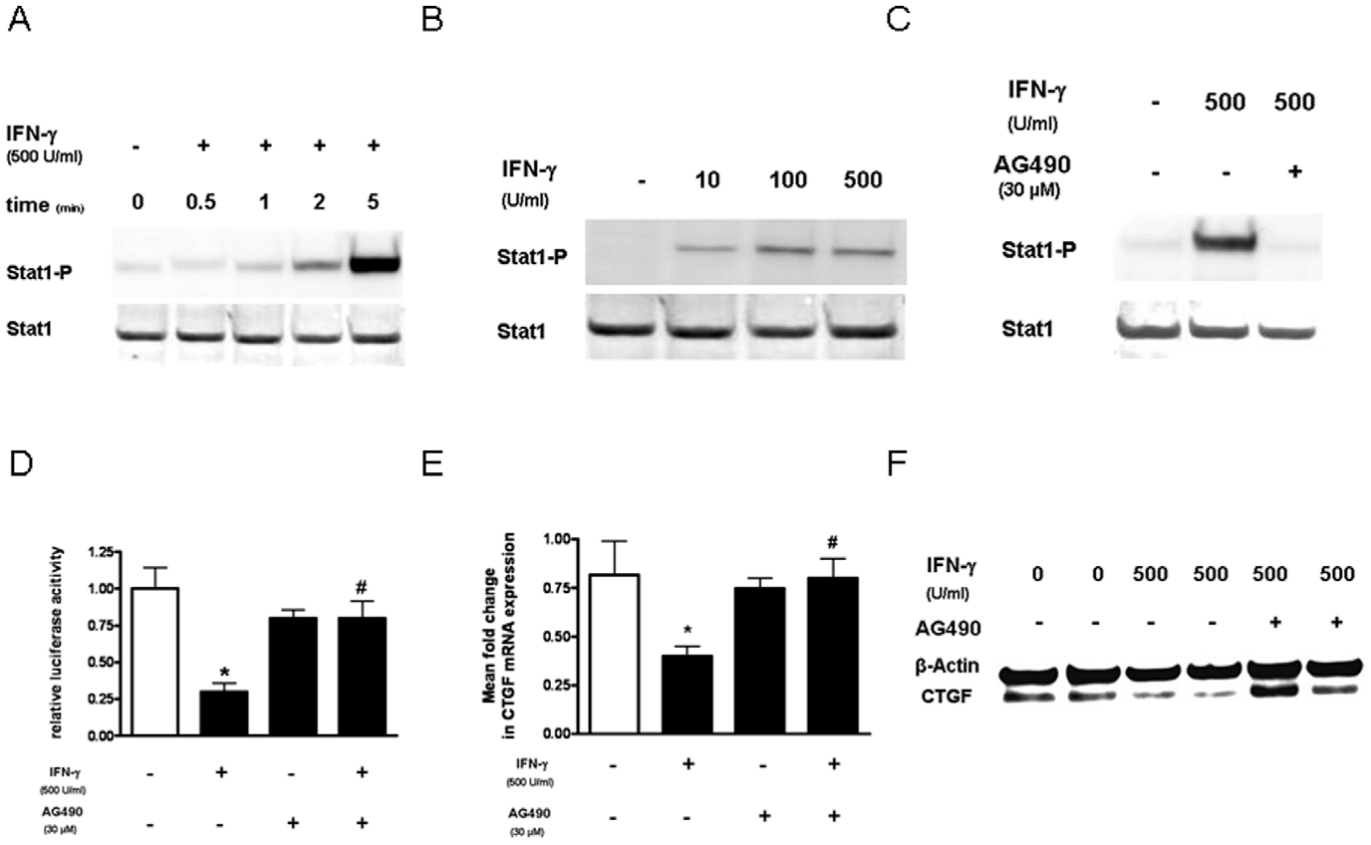
Effect of the Jak1-inhibitor AG-490 on IFN-γ induced inhibition of CTGF expression in LEC. LEC were incubated for different time points with 500 U/ml IFN-γ (A), with different concentrations of INF-γ for 5 min (B), or with or without the Jak-inhibitor AG-490 (30 µM) and INF-γ (500 U/ml) for 5 min (C). Stat1 phosphorylation and Stat1 expression were detected by immunoblotting with anti-phospho-Stat1 and anti-Stat1 antibodies. A representative of 3 independent experiments is shown. **D.** Luminometric analysis of CTGF-Luc reporter transfected LEC: LEC were incubated with medium alone (control) or with IFN-γ without or with the Jak-inhibitor AG-490 (30 µM). * p<0.05 vs. medium alone; # p<0.05 vs IFN-γ alone. **E+F.** LEC were incubated with IFN-γ with or without the Jak-inhibitor AG-490 (30 µM). CTGF mRNA expression compared to β-actin was measured by real-time PCR (**E**). CTGF and β-actin protein expression was determined by immunoblotting (**F**). Significant differences (p<0.05) compared with untreated cells are marked by *, compared with IFN-γ are marked with #. A representative of 3 independent experiments is shown (**F**).

In the next step we wanted to demonstrate that IFN-γ can activate Jak-Stat signaling in LEC. Phosphorylation of Stat1 was detected 1 min after addition of IFN-γ and further enhanced up to 5 min (Figure 3A). This effect of IFN-γ on Stat1 phosphorylation was dose-dependent, with a maximal effect at a concentration of 500 U/ml (Figure 3B), and could be inhibited by the Jak inhibitor AG-490 (Figure 3C). These results demonstrated that IFN-γ can induce Jak-Stat signaling in LEC, which could be blocked by the Jak-inhibitor AG-490.

In an attempt to define the role of the Jak/Stat signaling pathway in the down-regulation of CTGF by IFN-γ, we treated LEC with IFN-γ in the presence or absence of the Jak inhibitor AG-490, and analysed CTGF-promoter activity, -mRNA, and -protein expression. With all the three different methods, luciferase-Assay (Figure 3D), real-time quantitative PCR assay (Figure 3E), and western immunoblotting assay (Figure 3F), the inhibition of CTGF expression by IFN-γ in LEC could be partially blocked by the Jak inhibitor AG-490. These results demonstrated that the Jak-Stat signaling pathway was involved in the inhibitory effect of IFN-γ on CTGF-expression.

### Regulation of CTGF Expression by TNF-α in LEC

**Figure 4:**
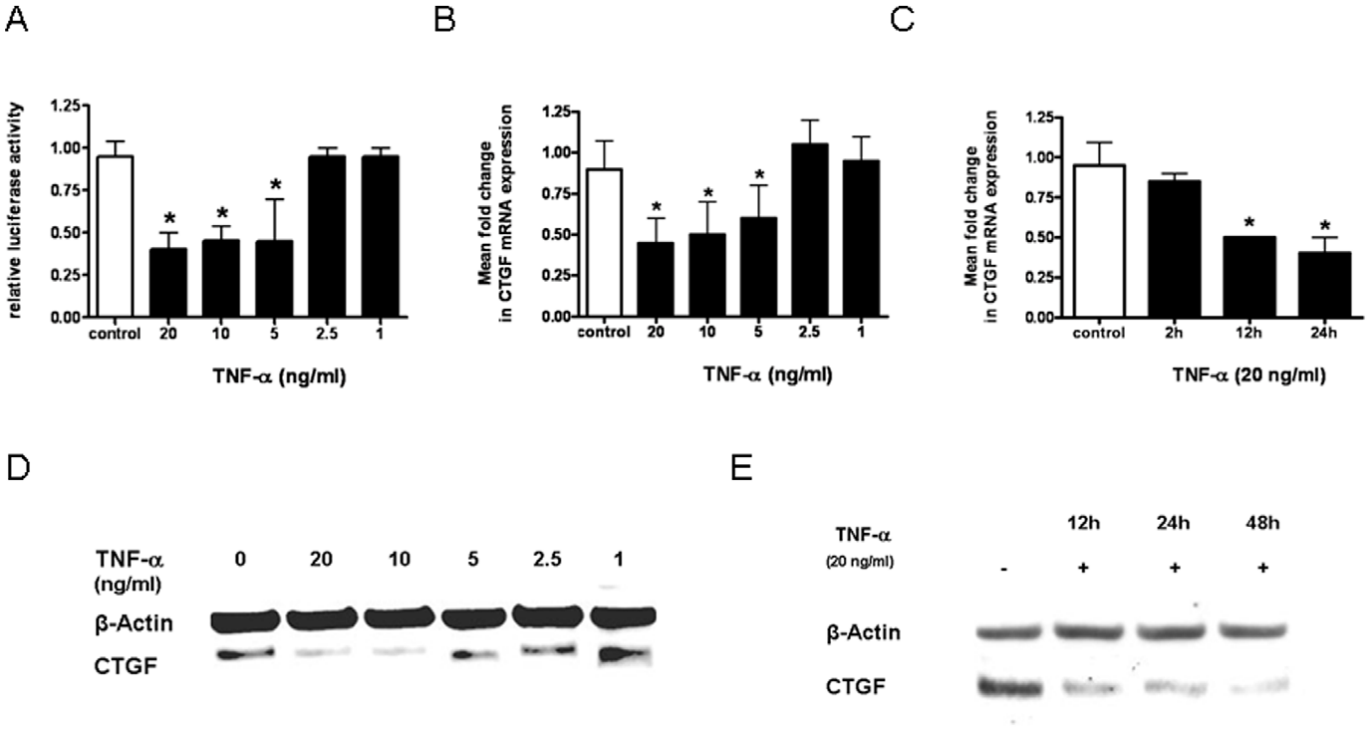
Effect of TNF-α on CTGF expression in LEC. **A.** Luminometric analysis of CTGF-Luc reporter transfected LEC: LEC were incubated with medium alone (control) or with decreasing concentrations of TNF-α. * p<0.05 vs medium alone. **B+C.** CTGF mRNA expression: LEC were incubated with different concentrations of TNF-α for 24 h (B) or for various duration with 20 ng/ml TNF-α (C). mRNA expression of CTGF normalized for β-actin were measured by real-time PCR. Significant differences (p<0.05) compared with untreated cells are marked by *. **D+E.** CTGF protein expression: LEC were incubated with different concentrations of TNF-α for 48 h (D) or with TNF-α (20 ng/ml) for different time periods (E). CTGF and β-actin expression was detected by immunoblotting. A representative of 3 independent experiments is shown.

At the next step we examined the effect of TNF-α on CTGF expression in LEC. At the promoter level, TNF-α inhibited CTGF-promoter activity in LEC in a dose dependent manner (Figure 4A). At a concentration of 20 ng/ml, TNF-α reduced CTGF-promoter activity 2.5-fold compared to untreated cells. At the transcriptional- and translational-levels we found a dose- and time-dependent reduction of CTGF mRNA with a maximal 2.5-fold inhibition after 24 h at a concentration of 20 ng/ml (Figure 4B+C) and of CTGF protein expression with a maximal decrease with 20 ng/ml TNF-α after 48 h (Figure 4D+E). Taken together, these data showed that TNF-α inhibited CTGF expression in LEC.

### Additive Effect of IFN-γ and TNF-α on CTGF Inhibition

**Figure 5:**
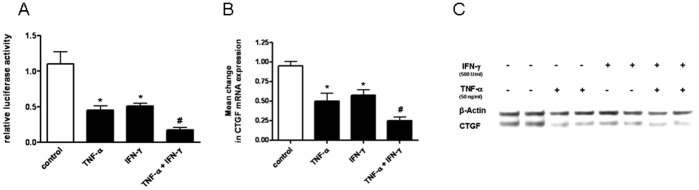
Additive effect of IFN-γ and TNF-α on the inhibition of CTGF expression. A. Luminometric analysis of CTGF-Luc reporter transfected HPMEC cells: LEC were incubated with medium alone (control), TNF-α, IFN-γ, or INF-γ and TNF-α. * p<0.05 vs medium alone; # p<0.05 vs INF-γ or TNF-α alone. **B+C.** LEC were incubated with TNF-α and/or IFN-γ. CTGF mRNA expression compared to β-actin was measured by real-time PCR (B). CTGF and β-actin protein expression was determined by immunoblotting (C). Significant differences (p<0.05) compared with untreated cells are marked by *, compared with INF-γ or TNF-α are marked with #. A representative of 3 independent experiments is shown (C).

In subsequent experiments we studied whether there was an additive effect of IFN-γ and TNF-α on the inhibition of CTGF expression in LEC. An additive effect on CTGF inhibition after the co-administration of IFN-γ and TNF-α could be detected on CTGF promoter activity (Figure 5A), on CTGF mRNA expression (Figure 5B), and on CTGF protein expression (Figure 5C). These results indicated that besides the inhibitory effect of IFN-γ and TNF-α alone, both cytokines had an additive effect on inhibition of CTGF expression in LEC when administered together.

### Additive Effect of IFN-γ and TNF-α on the Inhibition of LEC Proliferation

**Figure 6:**
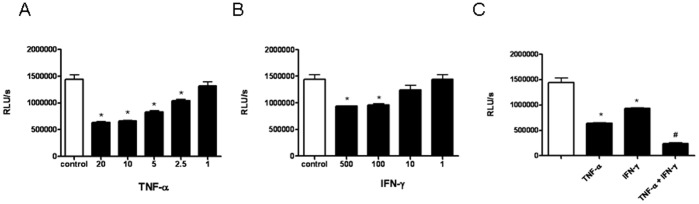
Additive effect of IFN-γ and TNF-α on the inhibition of LEC proliferation. Proliferation was assessed 5 days after the addition of TNF-α **(A)**, IFN-γ **(B),** or TNF-α + IFN-γ **(C)** at the indicated concentration. Values are means ± SEMs for 3 replicate experiments. Significant differences (p<0.05) compared with untreated cells are marked by *, compared with INF-γ or TNF-α are marked with #.

To investigate whether IFN-γ and TNF-α influenced the proliferative behaviour of LEC, the effect of IFN-γ and/or TNF-α on *in vitro* proliferation of human LEC was analysed (Figure 6A–C). Both TNF-α and IFN-γ reduced LEC proliferation in a dose-dependent manner. At the highest concentration tested, TNF-α (20 ng/mL) reduced proliferation of LEC to 43% (Figure 6A) and IFN-γ (500 U/ml) to 64% (Figure 6B). IFN-γ (500 U/ml) and TNF-α (20 ng/ml) co-operatively inhibited LEC proliferation below the levels of IFN-γ and TNF-α alone (16% versus 43% and 64%; Figure 6C). Compared with medium, IFN-γ and/or TNF-α had no influence on the percentage of viable LEC (data not shown). In summary, these results showed an additive effect of IFN-γ and TNF-α on inhibition of LEC proliferation.

### CTGF Silencing with shRNA in LEC

**Figure 7:**
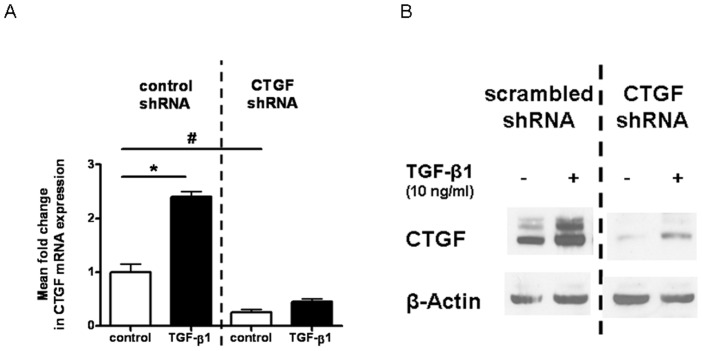
Effect of CTGF shRNA on basal and TGF-β1 mediated induction of CTGF mRNA- and protein-expression in LEC. A. Effect on CTGF mRNA level: Four days after downregulation of CTGF by specific shRNA LEC were cultured in serum reduced medium (2% FCS) for 24 h before treatment with TGF-ß1. RNA and protein was isolated after 24 h of incubation with 10 ng/ml TGF-ß1. CTGF RNA expression, compared to β-actin as control, was detected by RT-PCR (A). **B.** Effect on CTGF protein level: CTGF and ß-actin expression on protein level was detected by western immunoblotting (B). Significant regulations of CTGF by TGF-ß1 are marked by * (p<0.05), CTGF down-regulation by CTGF specific shRNA compared to control cells are marked by # (p<0.05).

To study the functional role of reduced CTGF expression in LEC, we generated lentiviral vector carrying CTGF-specific silencing shRNA. Stable transfection of LEC CTGF shRNA resulted in a decrease of CTGF mRNA basal level of up to 75% (Figure 7A) as well as of the protein level (Figure 7B) compared with non-specific scrambled control cells. The known induction of CTGF mRNA after TGF-β1 treatment was also inhibited by the CTGF shRNA (Figure 7A). Similar results were observed at the protein level for TGF-β1-induced CTGF expression (Figure 7B). These results demonstrated that production of CTGF in LEC was significantly knocked down by CTGF shRNA.

### Effect of CTGF Silencing on the Proliferation of LEC and the Inhibitory Effect of IFN-γ and TNF-α

**Figure 8:**
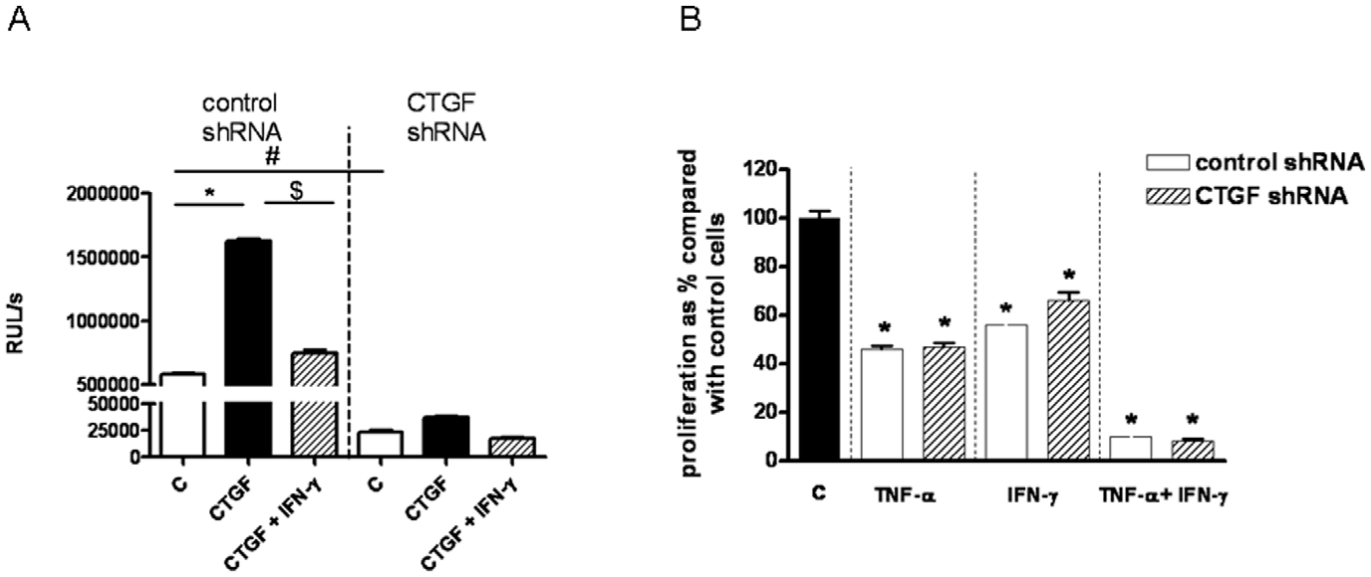
Effect of CTGF silencing on the proliferation of LEC and IFN-γ and TNF-α mediated inhibition. A. Proliferation of LEC, in which CTGF was down-regulated by CTGF shRNA transfection (right side; “CTGF shRNA”) and of control cells, which were transfected with non-specific scrambled shRNA (left side; “control shRNA”), was assessed after the addition of 100 ng/ml rCTGF with or without the addition of IFN-γ (500 U/ml) at 72 h. Values are means ± SEMs for 3 replicate cultures. Significant differences to response to rCTGF are marked by * (p<0.05), significant differences of proliferation between CTGF shRNA and non-specific scrambled shRNA transfected cells are marked by # (p<0.05), and significant differences of proliferation between CTGF-induced proliferation and CTGF-induced proliferation with the addition of IFN-γ are marked by $ (p<0.05). **B.** Proliferation of LEC, in which CTGF was down-regulated by CTGF shRNA transfection (lined column; “CTGF shRNA”) and of control cells, transfected with non-specific scrambled shRNA (white column; “control shRNA”), was assessed 5 days after the addition of TNF-α (20 ng/ml), IFN-γ (500 U/ml), or TNF-α+IFN-γ. Values are means ± SEMs for 3 replicate experiments and present as % compared with non stimulated cells (100%; black column). Significant differences between TNF-α and/or IFN-γ treated cells to control cells are marked by * (p<0.05).

Down-regulation of CTGF by shRNA transfection reduced the proliferation of HPMEC cells by 96% compared with non-specific scrambled control shRNA cells (Figure 8A). Furthermore, rCTGF-induced LEC proliferation was also reduced by the CTGF shRNA (1.7-fold increase) compared with non-specific scrambled control shRNA cells (2.6-fold increase) (Figure 8A). IFN-γ could antagonize CTGF-induced proliferation both in control and CTGF shRNA cells (Figure 8A).

In addition, we analysed the inhibitory effect of IFN-γ on the proliferation of CTGF-deficient LEC. No significant difference in IFN-γ-, TNF-α-, and combination of IFN-γ- and TNF-α-mediated proliferation inhibition was observed between control and CTGF shRNA cells. (Figure 8B). These results demonstrated that the inhibitory effect of IFN-γ and TNF-α on LEC proliferation was not mediated by down-regulation of CTGF.

## Discussion

IFN-γ is a well characterised immuno-modulatory and anti-fibrotic cytokine, whereas the role of IFN-γ in lung endothelial cell biology is less defined. The present study provided evidence that CTGF was negatively regulated by IFN-γ in LEC in a Jak/Stat signaling pathway-dependent manner. In addition, an additive effect of IFN-γ and TNF-α on inhibition of CTGF expression and cell proliferation could be found. CTGF silencing in LEC by transfection of CTGF shRNA reduced cell proliferation. However, CTGF silencing had no effect on IFN-γ and TNF-α mediated inhibition of LEC proliferation.

Evidence exists suggesting that IFN-γ could alter airway remodeling [15]–[18], but the involved molecular mechanisms are not known in detail. Concerning the fibrotic aspect, in a bleomycin-mouse model of lung fibrosis, IFN-γ down-regulated TGF-β gene expression and suppressed both the proliferation of fibroblasts and collagen synthesis [36]. A study of various forms of pulmonary fibrosis, including idiopathic pulmonary fibrosis, indicated that there may be a general impairment of the production of IFN-γ in patients with pulmonary fibrosis [37]. In patients with idiopathic pulmonary fibrosis, 6-month treatment with IFN-γ and low dose prednisolone has been reported to reduce the levels of transcription of both TGF-ß1 and CTGF, and to improve lung function and blood gases [38].

Concerning the vessels, IFN-γ can attenuate angiogenesis during wound healing [39]–[42]. Although an endogenous anti-angiogenic activity of IFN-γ has been described, the mechanism by which IFN-γ regulates angiogenesis is not well understood. According to our finding of an anti-proliferative effect of IFN-γ on LEC, anti-angiogenic effects of IFN-γ were previously described in endothelial cells in other tissues such as the lung [43]. IFN-γ caused growth inhibition and disrupted tube formation of human umbilical vein endothelial cells (HUVEC) [40]. These effects were also mediated by Stat1 [40], in agreement with our findings of IFN-γ influence on CTGF expression. Friesel et al. found that IFN-γ inhibits endothelial cell growth factor (ECGW)-induced cell proliferation of HUVEC with a concomitant change in endothelial cell morphology [43]. Furthermore, they demonstrated that the effects of IFN-γ on ECGF-induced proliferation correlated with a decrease in the number of ECGF receptors on the endothelial cell surface [43].

Besides the direct action of IFN-γ on endothelial cells, other studies provided evidence that IFN-γ indirectly mediates its angiostatic effect. IFN-γ could block or attenuate gene expression of several other known regulators of endothelial cell activation and angiogenesis, such as the induction of the angiostatic protein IP-10 (interferon-inducible protein-10) [44]. IFN-γ suppressed also VEGF-induced up-regulation of the angiogenetic protein angiopoetin-2 (Ang-2) or of the VEGF receptor VEGFR2 [40]. This suggests that up-regulation of angiostatic- or down-regulation of angiogenic-molecules by IFN-γ can be responsible for the anti-angiogenic activity of IFN-γ and influence the balance of angiogenic molecules in favor of the non angiogenic state. In relation to these observations, we found that IFN-γ can down-regulate CTGF expression in LEC.

Down-regulation of CTGF by IFN-γ has already been described by Fitzner et al. in pancreatic stellate cells (PCSs) and was also Stat1-dependent [45]. Also in keloid-derived fibroblasts and in rat kidney tubular epithelial cells (NRK52E) IFN-γ can reduce CTGF expression [46], [47]. Opposite effects of TGF-β and IFN-γ on trans-differentiation of myofibroblasts in human gingival cell cultures, with inhibition of CTGF expression by IFN-γ, was described by Sobral et al. [48]. However, no effect of IFN-γ on CTGF expression was found in cultured mesangial cells [49], which would suggest a cell type specific effect of IFN-γ on CTGF expression.

TGF-β is widely accepted as the most potent inducer of CTGF [8], while TNF-α was found to suppress CTGF expression in different cell types like bovine aortic endothelial cells or airway smooth muscle cells [29]–[31]. Our results confirmed CTGF inhibition by TNF-α also in LEC.

Beside a suppression of CTGF expression by TNF-α and IFN-γ alone in LEC, we could detect a new additive effect of IFN-γ and TNF-α co-administration on CTGF inhibition and on LEC-proliferation inhibition. Many genes which are regulated by IFN-γ are also regulated by TNF-α. Very often the induction was then synergistic, as described in the study by Stewart et al. [50]. Vascular endothelial platelet endothelial adhesion molecule-1 (PECAM-1) expression was synergistically reduced by TNF-α and INF-γ in endothelial cells [50]. In addition, endothelial cells treated simultaneously with TNF-α and IFN-γ demonstrated synergistic increases in the synthesis of the angiostatic CXCR3 chemokine compared with cells treated with TNF-α and IFN-γ alone [51]. Also a synergistic inhibition of proliferation of endothelial cells was observed when TNF-α and IFN-γ were simultaneously added to endothelial cells [51]. Furthermore, an additive effect of TNF-α and IFN-γ on the CXCL10 (IFN-γ-inducible protein-10 (IP-10)) expression in airway smooth muscle cells could be described [52]. It was postulated that the synergism between TNF-α and INF-γ involved an interaction between the TNF-α activated NF-κB signaling pathway and the transcription factors induced by IFN-γ like IRF-1 (interferon regulatory factor-1) [53]. Promotors of genes regulated by IFN-γ and TNF-α contain both an interferon-stimulated response element (ISRE) and an NF-κB-site. Cooperation of IFN-γ and TNF-α on CTGF inhibition in LEC and inhibition of LEC proliferation confirmed the relationship between these cytokines also in airway remodeling processes, as already described in other systems [41].

To study the functional effect of the downregulation of CTGF in LEC, HPMEC cells were stable transfected with CTGF specific shRNAs by a lentiviral system. Downregulation of CTGF could be shown at the RNA- and protein-level. CTGF silencing inhibited LEC proliferation, indicating that CTGF expression is also a crucial component for LEC proliferation. Proliferation of lung and dermal fibroblasts was also suppressed after CTGF-silencing [54], [55]. However, CTGF-silencing in LEC had no effect on IFN-γ induced Stat1 phosphorylation (data not shown) and the anti-proliferative effect of IFN-γ and TNF-α. Therefore, IFN-γ and TNF-α mediated CTGF inhibition was not responsible for the anti-proliferative effect described for IFN-γ and TNF-α.

Our data demonstrated that CTGF was negatively regulated by IFN-γ in LEC in a Jak/Stat signaling pathway-dependent manner. Down-regulation of the angiogenic molecule CTGF by IFN-γ could be responsible for the described anti-angiogenic activity of IFN-γ and influence the balance of angiogenic molecules in favor of the non angiogenic state. In addition, we found an additive effect of IFN-γ and TNF-α on CTGF inhibition, which underlines the relationship between these cytokines also in airway remodeling processes. The inverse correlation between IFN-γ and CTGF expression in LEC could mean that screwing the Th2 response to a Th1 response with IFN-γ production might be beneficial to avoid airway remodeling in asthma. Understanding the mechanisms involved in regulation of CTGF in LEC and downstream events mediated by CTGF could provide new therapeutic strategies for the treatment of special components of asthma, not addressed yet using the current therapeutic regimens.
